# Charging a Capacitor from an External Fluctuating Potential using a Single Conical Nanopore

**DOI:** 10.1038/srep09501

**Published:** 2015-04-01

**Authors:** Vicente Gomez, Patricio Ramirez, Javier Cervera, Saima Nasir, Mubarak Ali, Wolfgang Ensinger, Salvador Mafe

**Affiliations:** 1 Dept. de Física Aplicada, Universitat Politècnica de València, E-46022 València, Spain; 2 Dept. de Física de la Tierra i Termodinàmica, Universitat de València, E-46100 Burjassot, Spain; 3 Dept. of Material- and Geo-Sciences, Materials Analysis, Technische Universität Darmstadt, D-64287 Darmstadt, Germany; 4 Materials Research Department, GSI Helmholtzzentrum für Schwerionenforschung, Planckstrasse 1, D-64291, Darmstadt, Germany

## Abstract

We explore the electrical rectification of large amplitude fluctuating signals by an asymmetric nanostructure operating in aqueous solution. We show experimentally and theoretically that a load capacitor can be charged to voltages close to 1 V within a few minutes by converting zero time-average potentials of amplitudes in the range 0.5–3 V into average net currents using a single conical nanopore. This process suggests that significant energy conversion and storage from an electrically fluctuating environment is feasible with a nanoscale pore immersed in a liquid electrolyte solution, a system characteristic of bioelectronics interfaces, electrochemical cells, and nanoporous membranes.

In biological processes, nanoscale energy and information flows occur under significant environmental fluctuations. Enzymes operate in rapidly changing environments^1^,^2^ and the cell membrane is equipped with protein aqueous pores (ion channels^3^) which allow noisy electrical and biochemical signals to be transduced into net directional fluxes^4^,^5^,^6^. These facts suggest that artificial nanostructures could also perform useful information and energy transduction processes in highly fluctuating environments. The above question is of significance because external fluctuations are usually considered a nuisance, not an opportunity, in applications of nanoscale devices. In particular, although the correlation between the state of a system and its environmental fluctuations precludes the systematic harvesting of energy from *equilibrium* random fluctuations, this is not the case of *non-equilibrium* external fluctuations uncorrelated to the system state.

We study here the electrical rectification of large amplitude fluctuating potentials using a single nanopore immersed in an ionic aqueous solution. To provide an appropriate context, we summarize first other relevant studies. On the basis of previous work by Siwy *et al.*^7^,^8^, we have demonstrated the transduction of periodic electrical signals with zero time-averages into net average currents in the cases of a biomimetic nanopore functionalized with amino acid groups^9^, a cylindrical pore that can be blocked by a nanoparticle^10^, and the (wide pore) bacterial porin channel of *Escherichia coli* reconstituted on a planar lipid bilayer^5^. However, the scientific and technically relevant question of energy conversion and storage has not been addressed in the above cases. We experimentally and theoretically demonstrate here a simple procedure allowing the conversion of external random signals (electric potentials) into directional average fluxes (net currents) using a *single* conical nanopore. The net current obtained is used to charge an external load capacitor, demonstrating significant energy conversion and storage from a highly fluctuating external environment. Analogously to the case of the cell membrane ion channels, the nanopore immersed in a liquid electrolyte solution shows ionic selectivity and electrical rectification characteristics^5^. The experimental data can be described theoretically by simulating the capacitor charging and discharging processes.

Because we use a single biomimetic pore operating in an aqueous salt solution which is electrically coupled to a commercial capacitor, the reported experiments can be of relevance to bioelectronics, information processing, and energy conversion processes using electrochemical nanodevices^11^,^12^,^13^,^14^,^15^,^16^.

## Results and Discussion

**Figure 1:**
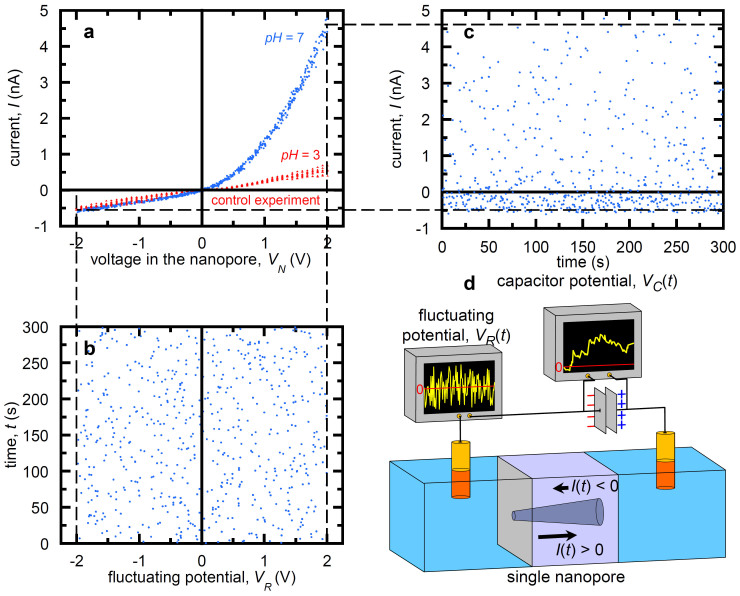
Scheme of the energy conversion and storage process using a single conical pore. (a) Steady-state current (*I*) - potential (*V_N_*) curves showing electrical rectification (*pH* = 7) and ohmic response (*pH* = 3, control experiment). (b) A randomly fluctuating electric potential *V_R_*(*t*) is externally applied using a voltage source. (c) The output electric current *I*(*t*) for *pH* = 7 gives a non-zero time average current <*I*>. (d) This net current <*I*> allows the charging of an external load capacitor connected in series to the nanopore. As the capacitor is charged, a potential difference (voltage) *V_C_*(*t*) which drives a reverse current opposing the charging process is set up.

Figure 1 schematically shows the rectification characteristics obtained using a single conical pore with a nanoscale tip radius which separates two identical 0.1 M KCl aqueous solutions. The pore is obtained by using asymmetric track-etching methods^17^,^18^ and its surface is functionalized with carboxylate residues that are ionized at *pH* = 7. The nanostructure has an asymmetric, negative fixed charge distribution along the axis which gives the electrical rectification observed in the steady-state current (*I*) – potential (*V_N_*) curve of the pore (Fig. 1a)^18^. A time (*t*)-dependent, randomly fluctuating electric potential *V_R_*(*t*) of amplitude *V*_0_ (white noise) is externally applied by means of a couple of Ag|AgCl electrodes immersed in the bathing solution (Fig. 1b). The output electric current *I*(*t*) shows a non-zero time average value <*I*> (Fig. 1c) as a result of the pore electrical rectification shown in Fig. 1a. The electric current *I*(*t*) through the pore shows no time delay with respect to *V_R_*(*t*) if the potential period is much longer than the relaxation time of the system. Because of the small pore volume involved, the above condition is approximately valid in nanopores^9^ and ion channels^5^ for the case of low frequency signals.

We use now the net current <*I*> to charge a commercial capacitor connected in series to the single nanopore (Fig. 1d), showing that steady-state capacitor voltages of the order of 0.2 V can be obtained for amplitudes *V*_0_ of the order of 1 V within a few minutes. Note finally that a control experiment for the same pore sample at the (low) *pH* = 3.0 shows an ohmic behavior (Fig. 1a) because the conical pore is in neutral form at this pH value. The absence of current rectification gives a zero net current in this case.

**Figure 2:**
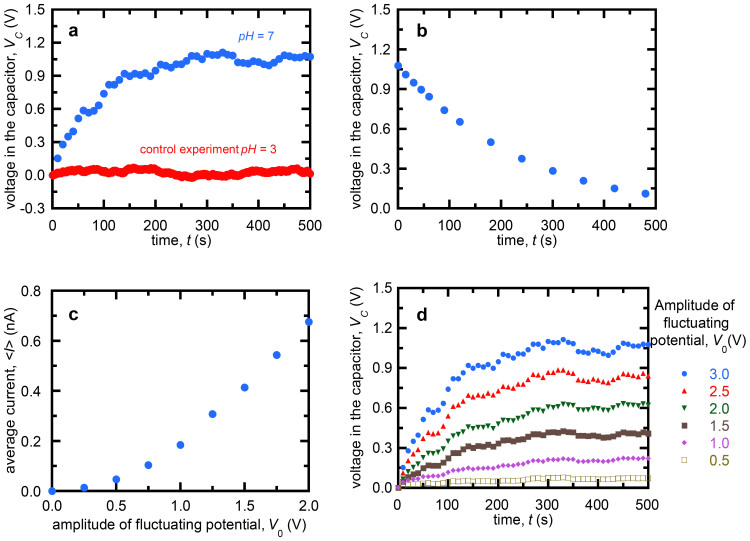
Experiments. (a) The capacitor potential *vs.* time charging for *pH* = 7 (top) and *pH* = 3 (bottom, control experiment) curves and (b) the discharging curve for pH = 7. In both cases the potential amplitude *V*_0_ = 3 V. (c) The average (net) current *vs*. the external potential amplitude. (d) The capacitor charging curves at different amplitudes of the potential. In all experiments the external load capacitance was *C* = 0.1 μF. The curves in Fig. 2d show identical time patterns because the input potential pulses are generated using the same scheme, scaling the single point values to obtain the desired amplitude. This procedure allows discarding the presence of internal noise sources due to the wiring, capacitor, and voltage source that might influence the phenomena studied.

Figure 2 shows typical charge (a) and discharge (b) curves measured with an external load capacitor of capacitance *C* = 0.1 μF and an input fluctuating potential of amplitude *V*_0_ = 3 V. The net current <*I*> as a function of *V*_0_ (c) and the capacitor charge curves at different values of *V*_0_ (d) are also shown. Fig. 2a shows that for the charged pore (*pH* = 7) at sufficiently long times *t*→*∞*, a maximum steady-state voltage *V_C_*(*∞*) is reached when the reverse current opposing the charging process is equal to the net charging current. The conical nanopore behaves then as a voltage-controlled current source: the maximum current corresponds to the initially uncharged capacitor, *V_C_*(*t* = 0) = 0, decreasing to zero when the capacitor reaches the voltage *V_C_*(*∞*). In the case of the neutral pore (*pH* = 3, control experiment in Fig. 2a), the capacitor voltage cannot reach significant average values because of the absence of current rectification (see Fig. 1a). Also, independent tests with other periodically fluctuating potentials (triangular and square zero-average potentials) were carried out, giving net currents and then significant capacitor charging.

When the external potential is disconnected, the capacitor begins to discharge following a characteristic exponential decay (Fig. 2b). As expected, increasing the amplitude *V*_0_ of the input signals leads to higher net currents <*I*>, as shown in Fig. 2c. The value of <*I*> does not depend on the random pulse lifetime because there is no time delay between the instantaneous current and the external potential (see Figs. 1a–c and Ref. 9 for details). The average currents <*I*> of Fig. 2c can be obtained directly from the experimental instantaneous currents *I*(*t*). Because the values of the input pulses are equally probable, these average currents should be approximately equal to those obtained by dividing the area limited by the *I*(*V_N_*) curve of Fig. 1a and the potential axis between −*V*_0_ and +*V*_0_ by the peak to peak value of this potential. We have checked that this is indeed the case, which gives further support to the validity of our assumptions. Note also that all curves in Fig. 2d show identical time patterns because the fluctuating signals are generated by the voltage source of Fig. 1d according to the same scheme. This procedure clearly suggests that the charging process arises only from the rectification of the input signal by the single nanostructure and is not significantly influenced by internal noise sources due to the electrical equipment.

Because of the increase of <*I*> with *V*_0_, the charging curves of Fig. 2d give steady capacitor voltages *V_C_*(*∞*) which increase with the input signal amplitude. Remarkably, *V_C_*(*∞*) is of the order of 1 V within a few minutes using a commercial capacitor. A measure of the charging process efficiency is given by the energy ratio 
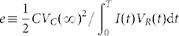
. From the experimental results of Fig. 2d, we can assume an effective total time *T = * 3*τ_ch_* for the capacitor voltage saturation, where *τ_ch_* is the characteristic charging time. The above energy ratio can then be obtained from the recorded experimental data and takes values between *e* = 0.016 (*V*_0_ = 0.5 V) and *e* = 0.071 (*V*_0_ = 3 V), which are small but not negligible. In this latter case, approximately 7% of the input energy from the external fluctuating signal can be recovered (i. e., 93% of the input energy is dissipated).

The single pore resistance in Fig. 1a is of the order of 1 V/1 nA = 1 GΩ and the capacitance of the load capacitor in Fig. 1d is 0.1 μF, giving characteristic times of the order of 10^9^ Ω × 10^−7^ F = 100 s, in agreement with the experimental curves of Fig. 2. In particular, the different characteristic times observed in the charging and discharging curves can be compared with the equivalent circuit times *τ_ch_* = *R_ch_C* (charging) and *τ_di_* = *R_di_C* (discharging), where *R_ch_* and *R_di_* are the effective single pore resistances for the charging and discharging processes. These times are of the order of 50 s and 200 s in Figs. 2a and 2b, respectively, in agreement with the different resistances of Fig. 1a obtained for forward (*V_N_* > 0) and reverse (*V_N_* < 0) pore polarization.

The results of Fig. 1a and 2a clearly show that it is the current rectification that gives the capacitor charging. As a consequence, this charging effect should be enhanced by increasing the pore rectification, which in turn depends on the pore geometry, the asymmetrical charge distribution, and the solution characteristics^9^,^15^,^18^. In particular, the absolute value of the positive and negative currents should decrease with the pore length and increase with the pore diameter. However, the net current that results from the difference between these absolute currents depends crucially on the pore rectification, which requires long pores and narrow tip diameters^15^.

**Figure 3:**
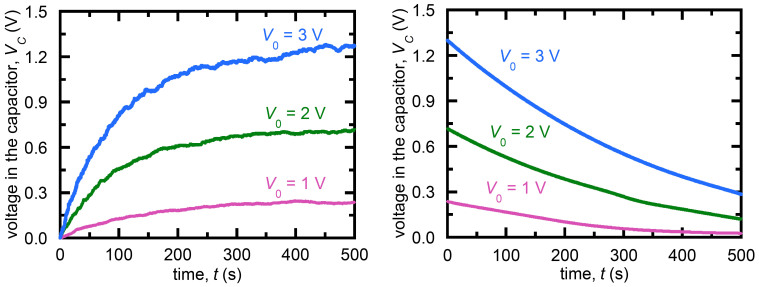
Simulations. (a) Simulated charging and (b) discharging curves for the potential amplitudes *V*_0_ = 1, 2, and 3 V.

The charging process can be simulated assuming that the nanopore is a potential dependent resistance connected in series to the capacitor. The whole circuit is fed with a (white noise) potential signal of amplitude *V*_0_. The equation describing the charging is d*V_C_*(*t*)/d*t* = *I*(*t*)/*C*, where *V_C_*(*t*) and *I*(*t*) are the instantaneous capacitor potential and the current through the circuit, respectively. We introduce the initial condition *V_C_*(*t* = 0) = 0, establish a constant time step Δ*t* for the potential *V_R_*(*t*) to take random values between −*V*_0_ and +*V*_0_, and update the capacitor voltage as *V_C_→V_C_ + I*(*V_N_*)Δ*t/C*, where *I*(*V_N_*) is the measured current (Fig. 1a) at the potential *V_N_* = *V_R_* – *V_C_*. This procedure leads to the final maximum voltage *V_C_*(*∞*). Then, we introduce *V_R_*(*t*) = 0 in the algorithm in order to simulate the discharging process. Figure 3 shows that the theoretical charging (Fig. 3a) and discharging (Fig. 3b) curves reproduce the observed behavior, which gives further support to the experimental data reported.

## Conclusion

A single asymmetric pore acting as a soft matter nanoscale version of the macroscopic solid-state diode allows charging a commercial capacitor to significant output potentials within a few minutes by rectifying an external, randomly fluctuating signal. The experimental data are correctly described by the theoretical simulations. The input signal amplitudes needed are dictated by the minimum voltage required for the nanostructure rectification to be significant and could then be decreased to 0.1 V in the case of (wide pore) biological ion channels (e.g*.*, the *OmpF* porin of *Escherichia coli* bacteria^5^) at the price of a decreased physico-chemical robustness. Also, the use of these channels could be useful to quantify the long-term accumulative effects of large amplitude and low frequency external signals.

The nanopore shows rectifying properties similar to those of wide pore biological ion channels^3^,^5^ and is immersed in the electrolyte solutions characteristic of nanopore-based sensors^19^,^20^, and bioelectronics interfaces^11^. The results suggest that physical mechanisms such as rectification can be exploited in the design of energy conversion and storage schemes based on soft matter nanostructures electrically coupled to conventional electronic elements.

## Methods

### Nanopore characteristics

Polyethylene terephthalate (PET) polymer foils (Hostaphan RN 12, Hoechst) of thickness 12 μm were irradiated with single swift heavy ions (Pb, U, and Au) having an energy of 11.4 MeV per nucleon at the linear accelerator UNILAC (GSI, Darmstadt) (PET). The single conical pore is obtained by using asymmetric track-etching methods described with detail elsewhere^17^,^18^. These methods give typical radii in the range 10–20 nm (cone tip) and 100–200 nm (cone basis). In our pore, the approximate radii were 11 nm and 155 nm, respectively, obtained from the steady-state current (*I*) – potential (*V_N_*) curve, where *V_N_* = *V_R_* – *V_C_*^18^. The etching process gives carboxylate residues on the pore wall surface which are ionized at *pH* = 7 in a 0.1 M KCl aqueous solution. Because of the approximately conical geometry, the nanostructure has an asymmetric (negative) fixed charge distribution^18^ along the axis which gives the electrical rectification observed in the *I* – *V_N_* curve of the pore (Fig. 1a). Also, additional control measurements were carried out at pH values low enough (*pH* = 3) for the pore to be in neutral form. The pH was controlled by a Crison GLP22 pH-meter before and after each measurement.

### Electrical measurements

The fluctuating electric potential is externally applied by means of a couple of Ag|AgCl electrodes immersed in the bathing solutions (Fig. 1d). Random pulse lifetimes in the range of 0.5 s were applied. The electrical rectification is due to the fact that the current entering the pore through the (narrow) tip opening experiences an electrical resistance lower than that entering the pore through the (wide) basis opening. Pore electrical measurements were recorded with one picoammeter/voltage source (Keithley 6487/E). The voltage across the commercial capacitor of capacitance *C* = 0.1 μF was measured with a multimeter (Keithley 2000/E). The average currents <*I*> of Fig. 2c were obtained directly from the recorded instantaneous currents *I*(*t*).

### Theoretical simulations

The charging process was simulated taking the nanopore as a potential dependent resistance connected in series to the capacitor. The whole circuit was fed with a (white noise) potential signal of amplitude *V*_0_. The equation describing the charging was d*V_C_*(*t*)/d*t = I(t)/C*, where *V_C_*(*t*) and *I*(*t*) were the instantaneous capacitor potential and the current through the circuit, respectively. We introduced the initial condition *V_C_*(*t* = 0) = 0 and established a (constant) time step Δ*t* = 0.1 s because this value is of the same order of magnitude as the experimental step used in the measurements. At each step Δ*t*, *V_R_*(*t*) took random values between −*V*_0_ and +*V*_0_ and the capacitor voltage was updated as *V_C_→V_C_ + I*(*V_N_*)Δ*t/C* where *I*(*V_N_*) is the experimental current of Fig. 1a. The simulations were carried out with different values of the time step Δ*t*. After completion of the charging process, a maximum voltage *V_C_*(*∞*) was reached. At this moment, we set *V_R_*(*t*) = 0 in the numerical algorithm to simulate the discharging process. All simulations were performed using a Python numerical code.

## Author Contributions

S.M. and P.R. designed the experiments, M.A., S.N. and W.E. fabricated and functionalized the nanopores, P.R. and V.G. performed the experiments, J.C. performed the simulations, P.R. and S. M. wrote the paper. All authors contributed to the concept of the paper and the discussion of the results.
